# Bioactive Peptides from* Angelica sinensis* Protein Hydrolyzate Delay Senescence in* Caenorhabditis elegans* through Antioxidant Activities

**DOI:** 10.1155/2016/8956981

**Published:** 2016-01-31

**Authors:** Qiangqiang Wang, Yunxuan Huang, Chuixin Qin, Ming Liang, Xinliang Mao, Shuiming Li, Yongdong Zou, Weizhang Jia, Haifeng Li, Chung Wah Ma, Zebo Huang

**Affiliations:** ^1^Guangdong Province Key Laboratory for Biotechnology Drug Candidates, School of Biosciences and Biopharmaceutics, Guangdong Pharmaceutical University, Guangzhou 510006, China; ^2^Research & Development Centre, Infinitus (China) Company Ltd., Guangzhou 510665, China; ^3^School of Pharmaceutical Sciences, Wuhan University, Wuhan 430071, China; ^4^Shenzhen Key Laboratory of Microbiology and Gene Engineering, College of Life Sciences, Shenzhen University, Shenzhen 518060, China

## Abstract

Since excessive reactive oxygen species (ROS) is known to be associated with aging and age-related diseases, strategies modulating ROS level and antioxidant defense systems may contribute to the delay of senescence. Here we show that the protein hydrolyzate from* Angelica sinensis* was capable of increasing oxidative survival of the model animal* Caenorhabditis elegans* intoxicated by paraquat. The hydrolyzate was then fractionated by ultrafiltration, and the antioxidant fraction (<3 kDa) was purified by gel filtration to obtain the antioxidant* A. sinensis* peptides (AsiPeps), which were mostly composed of peptides with <20 amino acid residues. Further studies demonstrate that AsiPeps were able to reduce the endogenous ROS level, increase the activities of the antioxidant enzymes superoxide dismutase and catalase, and decrease the content of the lipid peroxidation product malondialdehyde in nematodes treated with paraquat or undergoing senescence. AsiPeps were also shown to reduce age pigments accumulation and extend lifespan but did not affect the food-intake behavior of the nematodes. Taken together, our results demonstrate that* A. sinensis* peptides (AsiPeps) are able to delay aging process in* C. elegans* through antioxidant activities independent of dietary restriction.

## 1. Introduction

Reactive oxygen species (ROS) are natural by-products of cellular metabolism and play a role in cell signaling and cellular homeostasis. Excessive accumulation of ROS, however, causes damage to proteins, lipids, and nucleic acids and leads to cell injury and death, which is recognized to contribute to aging process and age-related diseases [1, 2]. Under normal conditions, an organism keeps a dynamic balance between ROS production and scavenging, in which the redundant ROS is largely antagonized by intricate antioxidation systems, including the antioxidant enzymatic scavengers superoxide dismutase (SOD) and catalase (CAT) [1]. Nonetheless, certain external as well as internal factors such as heat shock, toxins, and oxidants can increase ROS level sharply and make the antioxidation defense systems in a weak position, leading to disruption of the balance between generation and elimination of ROS and consequently progression of diseases and even eventual death [3, 4]. Therefore, antioxidants capable of scavenging excessive ROS may help maintain oxidative homeostasis and prevent related damages.

A number of natural products such as resveratrol [5], epigallocatechin gallate [6], salidroside [7], and* Ginko biloba* extract EGb 761 [8] are found to have antioxidant effect. In recent years, antioxidant peptides derived from food and herbal sources have, as natural and nontoxic products, drawn more and more attention. These bioactive peptides include specific protein fragments of 2–20 amino acid residues, which are concealed in the parental proteins with their biological activities emerged after release by enzymatic hydrolysis [9, 10]. Medicinal plants, especially edible herbs, are shown to be an important source of bioactive peptides with potent antioxidant activities [11–13].

*Angelica sinensis* (Oliv.) Diels, a popular traditional Chinese medicine first noted in Shen Nong's Herbal Classics (~110 BC), has been widely used for tonifying blood to promote blood circulation and regulate menstruation. It has also been used as health food for women's care in Europe and America [14]. Recent studies have shown that polysaccharides, among other products, from* A. sinensis* have antioxidant and neuroprotective functions [15], but similar activities have not been addressed on peptides from* A. sinensis*. Using the nematode* Caenorhabditis elegans*, a powerful model organism for aging and neurobiology studies, we investigated the antioxidant and anti-aging activities of peptide preparations derived from* A. sinensis* roots, including their effect on oxidative survival, ROS levels, antioxidant enzyme activities, lipid peroxidation product content, age pigments content, and lifespan.

## 2. Materials and Methods

### 2.1. Chemicals and Materials

Trypsin from bovine pancreas (≥2,500 IU/mg) and methyl viologen dichloride were purchased from Aladdin Co. (Shanghai, China). Papain (6,000 USP U/mg) was purchased from Solarbio Co. (Beijing, China). 2,7-Dichlorofluorescein diacetate (DCFH-DA) and 5-fluoro-2′-deoxyuridine (FUdR) were bought from Sigma-Aldrich Co. (St. Louis, MO, USA). Sephadex G-25 (medium) was obtained from GE Healthcare Co. (Uppsala, Sweden). Ammonium sulfate (analytical grade) was obtained from Guangzhou Chemical Reagent Factory (Guangzhou, China). SOD, CAT, and malondialdehyde (MDA) assay kits were purchased from Beyotime (Haimen, China). BCA Protein Assay Kit was bought from Pierce Chemical Co. (Rockford, IL, USA).

### 2.2. Preparation of* Angelica sinensis* Protein Isolates

Sliced* A. sinensis* roots were purchased from Beijing Tongrentang Yinpian Co., Ltd. (Bozhou, China) and ground into powder. The protein isolates were prepared essentially as described [16]. Briefly, the powder was soaked in 20 mM Tris-HCl buffer at pH 7.4 for 12 h and the suspension was centrifuged at 5,000 ×g for 30 min. The supernatant was collected and then replenished with solid ammonium sulfate at 4°C to reach 80% of saturation. After 4 h, the solution was centrifuged at 5,000 ×g for 30 min at 4°C, and the pellet was collected, resuspended in water, and dialyzed (molecular weight cut-off 3,500 Da) to remove salt and small molecule impurities. The liquid was then freeze-dried as* A. sinensis* protein isolates and stored at 4°C.

### 2.3. Enzymatic Hydrolysis

The* A. sinensis* protein isolates were hydrolyzed by trypsin and papain as follows. Approximately 10 g of the above protein isolates was dissolved in 200 mL of deionized water and the pH was adjusted to 8.0 with 0.5 M NaOH. After adding 2.4 mL of trypsin solution (6,250 IU/mL), the enzymatic hydrolysis was performed at 45°C in a water bath for 4 h. The solution was then heated to 60°C and the pH was adjusted to 6.0 with 12 M HCl to inactivate the enzyme. After adding 0.5 mL of papain (30,000 USP U/mL), the solution was incubated at 60°C for 4 h. The hydrolysis was terminated by heating in boiling water for 10 min. The solution was then freeze-dried as* A. sinensis* protein hydrolyzate (AsPH).

### 2.4. Isolation and Purification of Peptides

AsPH (20 mg/mL) was fractionated by centrifugal ultrafiltration (Vivaspin 20, Sartorius) using molecular weight (MW) cut-off of 5 kDa and 3 kDa, respectively, and 3 fractions were obtained: AsPH-F1 (MW > 5 kDa), AsPH-F2 (3 kDa < MW < 5 kDa), and AsPH-F3 (MW < 3 kDa). After bioactivity test (see below), the bioactive AsPH-F3 (20 mg/mL) was further separated on a Sephadex G-25 gel filtration chromatography column (1.2 cm × 150 cm), which was eluted with deionized water at a flow rate of 1 mL/min and monitored at 280 nm with a 785A UV/VIS detector (Perkin Elmer Co., Norwalk, CT, USA). The purified fraction was collected and freeze-dried as AsiPeps.

### 2.5. Identification of Peptides by LC-MS/MS Analysis

To identify peptide sequence, the peptide samples were separated by reverse phase nanoflow HPLC and analyzed by tandem mass spectrometry. Briefly, 2 *μ*g of peptides was redissolved in solvent A (water/acetonitrile/formic acid, 98 : 2 : 0.1, v/v/v) and loaded on ChromXP C18 (3 *μ*m, 120 Å) nanoLC trap column. The online chromatography separation was employed on the Eksigent nanoLC-Ultra*™* 2D System (AB SCIEX, Concord, Ontario, Canada). Desalting procedure was carried out at 2 *μ*L/min for 10 min with 100% solvent A. Then, a linear gradient of 5–35% solvent B (water/acetonitrile/formic acid, 2 : 98 : 0.1, v/v/v) over 50 min was used on an analytical column (75 *μ*m × 15 cm C18, 3 *μ*m, 120 Å, ChromXP Eksigent). LC-MS/MS analysis was performed with a TripleTOF 5,600 System (AB SCIEX, Concord, Ontario, Canada) fitted with a Nanospray III source (AB SCIEX, Concord, Ontario, Canada). Data were acquired using an ion spray voltage of 2.5 kV, curtain gas of 30 PSI, nebulizer gas of 5 PSI, and an interface heater temperature of 150°C. The MS was operated with TOF-MS scans. For the information dependant acquisition, survey scans were acquired in 250 ms and as many as 25 product ion scans (80 ms) were collected if exceeding a threshold of 150 counts/s and with a +2 to +5 charge-state. A rolling collision energy setting was applied to all precursor ions for collision-induced dissociation. Dynamic exclusion was set for 1/2 of peak width (~12 s). All raw data files (^*∗*^.wiff) were collectively searched with ProteinPilot Software v. 4.5 (AB SCIEX, Foster City, California, USA) against a dicotyledonous plant protein database. The detected protein threshold (unused ProtScore) was set to 1.3 (95% confidence). Peptides were filtered at 1% false discovery rate.

### 2.6. Strains and Maintenance

Both* Caenorhabditis elegans* (wild-type N2) and* Escherichia coli* (OP50 and NA22) strains were obtained from the* Caenorhabditis* Genetics Center (University of Minnesota, USA). All experiments were performed at 20°C unless otherwise stated.

### 2.7. Paraquat Survival Assay

The oxidative survival assay was performed as described previously using paraquat [7]. Synchronized L1 larvae were incubated for 42 h, and then 75 *μ*g/mL FUdR was added. After further incubation for 24 h, the nematodes reached young adulthood and were transferred to 96-well plates (~20 nematodes/well; >100 nematodes for each treatment) containing* E. coli* NA22 (OD_570 nm_ = 0.5) and peptide samples. After incubation for another 24 h, the nematodes were exposed to 70 mM paraquat. The numbers of live and dead nematodes were scored microscopically every 12 h based on their movement and shape; before counting, the plate was gently vibrated to stimulate movement of the animals.

### 2.8. Determination of ROS Levels

To assess ROS levels in paraquat-stressed nematodes, synchronized young adults were incubated with or without the peptide samples for 24 h before paraquat was added at 2 mM final concentration [17]. After treatment with paraquat for 2 days, the nematodes were harvested and washed for 3 times with M9 buffer to remove remaining paraquat and bacteria. The determination of ROS levels was performed as described previously [18] with modifications. The nematodes were dispensed into black 96-well plates by a COPAS Biosort instrument (Union Biometrica, Inc., Holliston, MA, USA), which is a flow cytometer capable of sorting and dispensing individual nematodes as well as measuring their physical and optical parameters [19]. After the dispense (100 animals per well; 1,000 animals for each treatment), the fluorescent probe DCFH-DA was added at a 50 *μ*M final concentration and the plates were incubated at 20°C for 14 h. Then ROS-related 2,7-dichlorofluorescein (DCF) fluorescence was measured at room temperature by a Fluoroskan Ascent FL plate reader (Thermo Electron Co., Waltham, MA, USA) at excitation 485 nm and emission 520 nm. For determination of ROS levels in senescent nematodes, synchronized young adults with 100 *μ*g/mL of ampicillin (but without paraquat) were incubated with or without the peptides to Day 10 of adulthood, and ROS was determined as above.

### 2.9. Determination of Antioxidant Enzyme Activity and MDA Content

Antioxidant indexes were determined as previously described [7] with minor modifications. Briefly, about 4,000 nematodes were washed three times, transferred into an Eppendorf tube, and suspended with 600 *μ*L of lysis buffer. The samples were sonicated in ice bath to obtain homogenate. After centrifugation (13,000 ×g, 5 min, 4°C), the supernatant was collected for measurement of SOD activity, CAT activity, MDA content, and protein content using commercial chemical assay kits, respectively. The values of enzyme activities and MDA content were normalized by protein content.

### 2.10. Age Pigments Accumulation and Lifespan Analyses

The accumulation of age pigments was assessed as described [20]. Briefly, Day-10 adult nematodes with or without peptide treatment were dispensed to black 96-well plates (100 nematodes per well) by COPAS Biosort, and the fluorescence of age pigments was determined by Fluoroskan Ascent FL microplate reader with 355 nm excitation and 460 nm emission. The lifespan assay was performed in 96-well plates using liquid culture as previously described [21]. The number of live nematodes was scored microscopically every 2 days based on their mobility, shape, and pharyngeal pumping until all the nematodes died [22].

### 2.11. Determination of Extinction, Time of Flight, and Pharyngeal Pumping Rate

Day-3 adult nematodes with or without peptide treatment were used for extinction and time of flight (TOF) and pharyngeal pumping rate determination. Extinction and TOF were determined by COPAS Biosort as previously described [23] using approximately 200 nematodes in each group. The pharyngeal pumping rate was counted manually under a dissecting microscope in one minute [24]. Over 20 animals were randomly selected in each group to score for the pumping rates.

### 2.12. Acquisition of Visual Images

All images were captured by ImageXpress Micro System (Molecular Devices, Sunnyvale, CA, USA) with a ×10 objective. Specifically, the fluorescent images of DCF were taken with a FITC excitation and emission filter setup, while the bright-field images were taken with 20% transmitted light filter setup.

### 2.13. Statistical Analysis

GraphPad Prism version 5.01 for Microsoft Windows (GraphPad Software, San Diego, CA, USA) was used for statistical analysis and one-way analysis of variance (ANOVA) was performed in multiple group comparisons.* C. elegans* survival and lifespan curves were analyzed by Kaplan-Meier method and log-rank test. Probability values of *p* < 0.05 were considered as statistically significant. All experiments were performed at least three times.

## 3. Results and Discussion

### 3.1. Increase of Oxidative Survival by* A. sinensis* Protein Hydrolyzate in* C. elegans*

**Figure 1:**
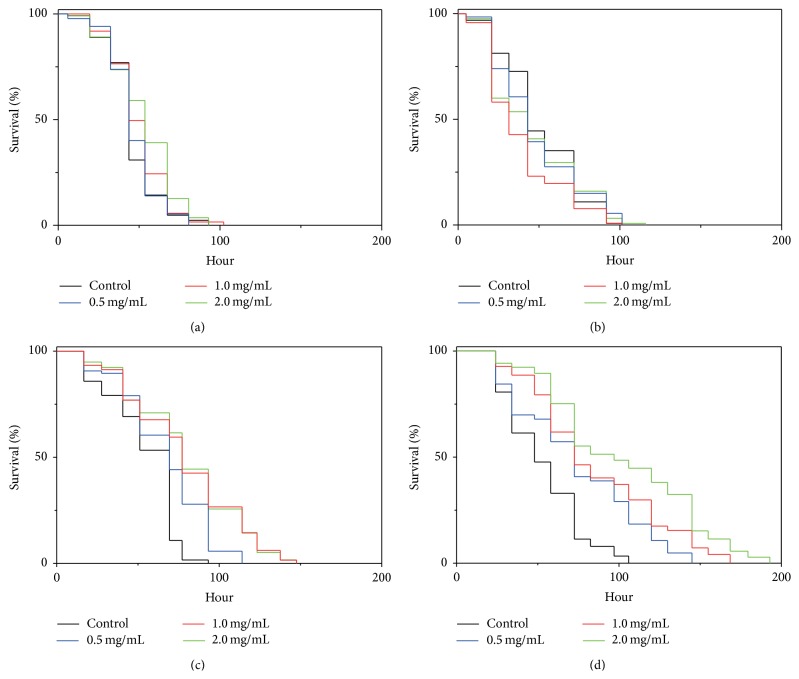
Effect of protein hydrolyzates on survival rates of paraquat-treated* C. elegans*. Young adult nematodes were pretreated with protein hydrolyzates at indicated concentrations at 20°C for 24 h prior to exposure to 70 mM paraquat, and the survival rates were scored every 12 h. (a)–(d) Representative Kaplan-Meier survival curves of nematodes treated with protein hydrolyzates from* Panax ginseng*,* Fallopia multiflora*,* Astragalus membranaceus*, and* Angelica sinensis*, respectively.

**Figure 2:**
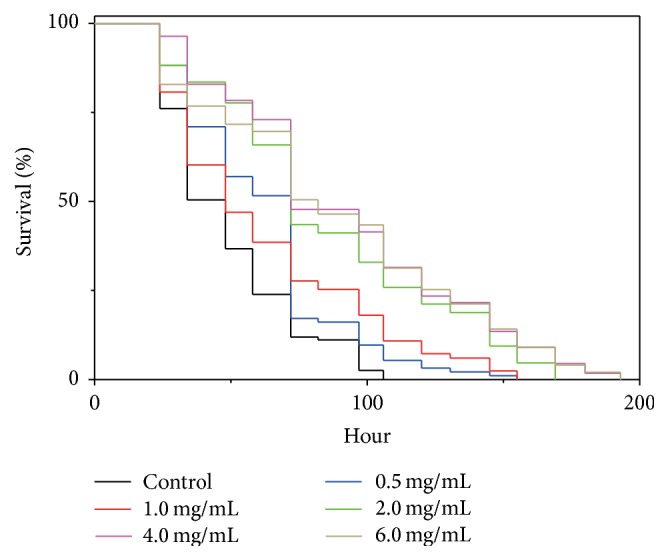
Effect of* A. sinensis* protein hydrolyzate concentration on survival rates of paraquat-treated* C. elegans*. The nematodes were treated with* A. sinensis* protein hydrolyzate and paraquat as in Figure 1. Representative Kaplan-Meier survival curves are shown for the nematodes treated with a series of concentrations of* A. sinensis* protein hydrolyzate.

Excessive generation of intracellular ROS will damage cellular structure and function, leading to aging and diseases of organisms [25–27]. Most studies on antioxidant peptides, however, are focused on* in vitro* scavenging activities against hydroxyl, superoxide, and other radicals [9–12], despite the fact that discrepancy exists between* in vitro* and* in vivo* antioxidant capacities of compounds [28–30]. Paraquat, a superoxide generator, induces an acute oxidative stress and causes rapid death of* C. elegans* at high doses [31]. Therefore, we performed* in vivo* oxidative survival assay to screen for antioxidant peptides using* C. elegans* models intoxicated by 70 mM paraquat. Preliminarily, we prepared protein hydrolyzates from >40 different medicinal plants and marine organisms. As an example, the effect of protein hydrolyzates from herbal plants* Panax ginseng*,* Fallopia multiflora*,* Astragalus membranaceus*, and* Angelica sinensis* on the survival rate of paraquat-treated nematodes is presented in Figure 1, in which* A. sinensis* protein hydrolyzate (AsPH) showed noteworthy capacity of improving survival of the nematodes under increased oxidative stress. To further determine the effective doses of AsPH, the nematodes were preincubated with a series of AsPH concentrations and then treated with 70 mM paraquat. As shown in Figure 2, AsPH increased the oxidative survival of nematodes in a dose-dependent manner with concentrations of >2 mg/mL being more potent.

### 3.2. Isolation and Identification of Antioxidant* A. sinensis* Peptides

**Figure 3:**
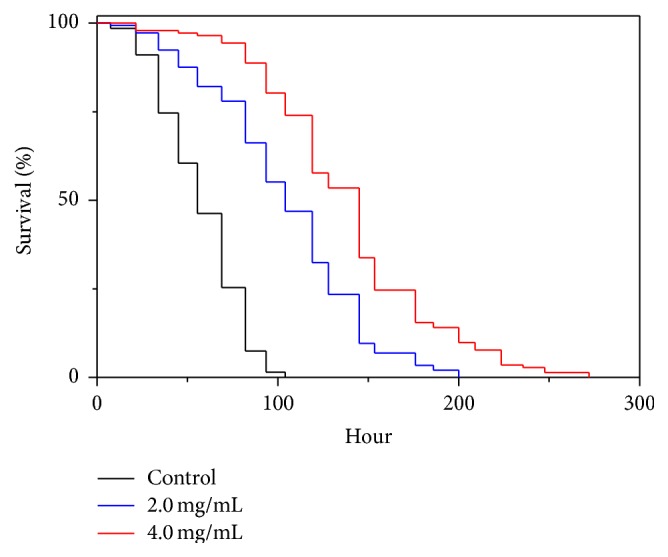
Effect of* A. sinensis* peptides on survival rates of paraquat-treated* C. elegans*. The nematodes were treated with* A. sinensis* peptides (AsiPeps) and paraquat as in Figure 1 and representative Kaplan-Meier survival curves are shown from three independent experiments.

Ultrafiltration, which is an effective approach to fractionate and concentrate proteins and peptides, was used to fractionate AsPH based on molecular weight, and 3 fractions of protein hydrolyzate were obtained, that is, AsPH-F1 (MW > 5 kDa), AsPH-F2 (3 kDa < MW < 5 kDa), and AsPH-F3 (MW < 3 kDa). The antioxidant capacities of the fractions were tested using the above paraquat resistance assay in* C. elegans*, which showed that AsPH-F3 had higher antioxidant activity than the other 2 fractions (data not shown). AsPH-F3 was, therefore, further purified by gel filtration chromatography on a Sephadex G-25 column, and the eluate was collected and freeze-dried as* A. sinensis* peptides (AsiPeps), which was subjected to paraquat resistance assay as above and showed strong antioxidant capacity at 2.0 and 4.0 mg/mL (Figure 3). Using LC-MS/MS, 27 peptides, consisting of 6–19 amino acid residues, were identified from AsiPeps; another peptide was composed of 26 amino acids (Table 1). This is in agreement with previous studies on natural peptides showing that most antioxidant peptides are <20 amino acid residues [10, 32–34]. For example, a 13-amino-acid peptide (DNYDNSAGKWWVT) from hydrolyzed cocoa by-product is shown to protect* C. elegans* against oxidative stress [35].

### 3.3. Decrease of ROS Level by* A. sinensis* Peptides in* C. elegans* under Oxidative Stress

**Figure 4:**
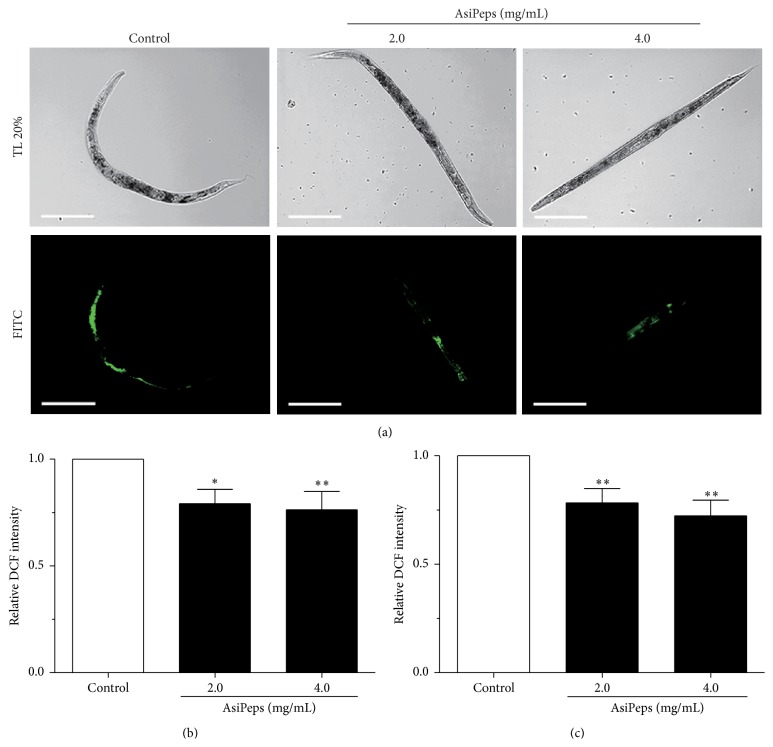
Effect of* A. sinensis* peptides on ROS levels of* C. elegans*. (a) Representative bright-field and corresponding fluorescent images of nematodes captured by ImageXpress Micro System with 20% transmitted light and FITC filter cubes, respectively. The nematodes were pretreated with* A. sinensis* peptides (AsiPeps) for 24 h prior to exposure to 2 mM paraquat. Scale bars: 200 *μ*m. (b) Effect of* A. sinensis* peptides (AsiPeps) on ROS level in nematodes exposed to 2 mM paraquat. (c) Effect of* A. sinensis* peptides (AsiPeps) on ROS level in nematodes undergoing senescence (Day 10). The DCF fluorescence intensity in (b) and (c) was detected by a microplate reader at 485 nm excitation and 520 nm emission, and the data are shown as mean ± SD of three independent experiments. ^*∗*^ *p* < 0.05; ^*∗∗*^ *p* < 0.01.

Since the* A. sinensis* peptides (AsiPeps) showed strong resistant capacity against high dose of paraquat (70 mM), we further tested its effect against paraquat toxicity at a low dose (2 mM), which generated a gentle oxidative stress but did not affect the survival rate of* C. elegans* during the test. The nematodes were preincubated with AsiPeps and then exposed to 2 mM paraquat before ROS determination. After incubation with the fluorescent probe DCFH-DA, the DCF fluorescence in the nematodes, as revealed by ImageXpress Micro System, was reduced after treatment with both 2.0 and 4.0 mg/mL of AsiPeps as compared to the controls, suggesting decreased ROS levels (Figure 4(a)). Further quantification by DCF fluorescence assay using microplate reader also demonstrates that the ROS levels of paraquat-exposed nematodes were reduced for >20% after AsiPeps treatments (Figure 4(b)). These data suggest that the capacity of AsiPeps to decrease endogenous ROS accumulation contributes to their protective effect against oxidative damage.

### 3.4. Increase of Antioxidant Enzyme Activities and Reduction of MDA Content by* A. sinensis* Peptides in* C. elegans* under Oxidative Stress

The antioxidant enzyme system is one of the most important lines of defense to confront oxidative stress and scavenge intracellular ROS in organisms. Accordingly, regulation of the antioxidant enzyme activities may contribute to increased survival rate of* C. elegans* under oxidative stress [36, 37]. On the other hand, malondialdehyde, one of the end-products of lipid peroxidation, is an important biomarker for oxidative stress [38]. Therefore, we investigated the effect of AsiPeps on the activity of antioxidant enzymes SOD and CAT and the content of MDA. As shown in Table 2, the SOD and CAT activities were increased, while the MDA content was decreased in the nematodes pretreated with 2.0 and 4.0 mg/mL AsiPeps, as compared to the control nematodes treated only with 2 mM paraquat. These data demonstrate that AsiPeps are able to improve the antioxidant defense system of nematodes through regulation of antioxidant enzyme activities and lipid peroxidation.

### 3.5. Effect of* A. sinensis* Peptides on ROS Level, Antioxidant Enzyme Activities, and Malondialdehyde Content in* C. elegans* Undergoing Senescence

Aging process is widely recognized to be associated with the degree of oxidation. Therefore, we determined the effect of* A. sinensis* peptides on ROS level, SOD and CAT activities, and MDA content in senescent nematodes. As shown in Figure 4(c), at Day 10 after adulthood, the ROS level was decreased >20% in nematodes treated with 2.0 and 4.0 mg/mL of AsiPeps as compared to the control nematodes without peptide treatment. The SOD and CAT activities were also increased, while the MDA content was reduced in the Day-10 nematodes treated with 4.0 mg/mL AsiPeps as compared to the controls (Table 3). These data demonstrate that the* A. sinensis* peptides are able to increase the antioxidant capacity of nematodes undergoing senescence.

### 3.6. Reduction of Age Pigments Accumulation and Extension of Lifespan by* A. sinensis* Peptides in* C. elegans*

**Figure 5:**
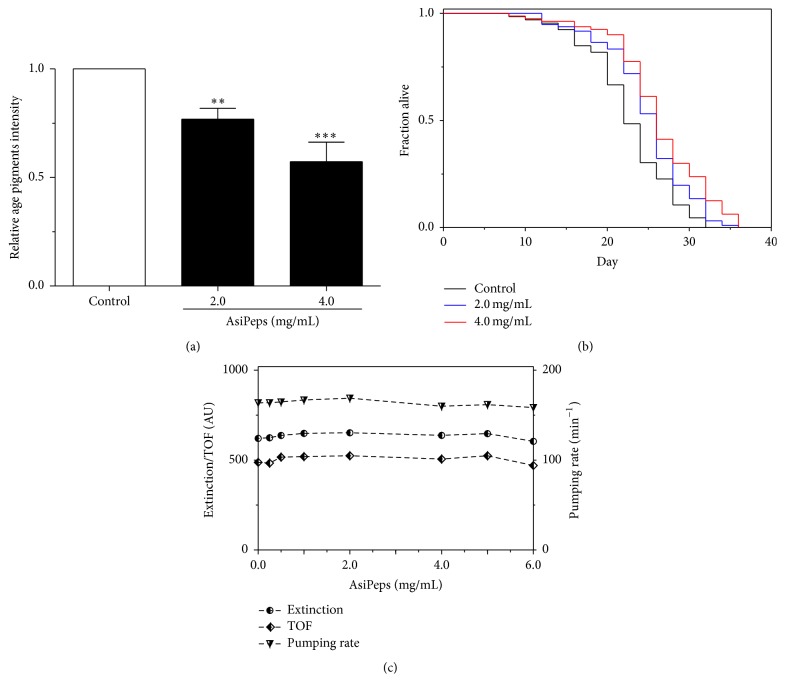
Effect of* A. sinensis* peptides on age pigments content, lifespan, and food-intake behavior of* C. elegans*. (a) Relative level of intestinal age pigments in senescent nematodes treated with* A. sinensis* peptides (AsiPeps). The fluorescence intensity of age pigments was detected at Day 10 by a microplate reader at 355 nm excitation and 460 nm emission. Data are shown as mean ± SD of three independent experiments. ^*∗∗*^ *p* < 0.01; ^*∗∗∗*^ *p* < 0.001. (b) Lifespan of nematodes with or without AsiPeps treatment. Representative Kaplan-Meier survival curves are shown from three independent experiments (>100 animals in each group). (c) Extinction, TOF, and pharyngeal pumping rate of the nematodes with or without AsiPeps treatment. AU: arbitrary units.

As shown above, the* A. sinensis* peptides (AsiPeps) can improve the antioxidant capability of* C. elegans* both under oxidative stress and at senescent stage, suggesting that AsiPeps are likely to play a role in the delay of aging process. Age-related autofluorescent age pigments, including lipofuscin and advanced glycation end-products, are widely regarded as biomarkers of aging, and their accumulation is shown to be inversely correlated with longevity [39]. In* C. elegans*, the age pigments are accumulated in the intestine over time and increase significantly from the 10th to 15th days after hatching at 20°C and can be quantified using fiber optic-coupled spectrofluorimetry [19]. Therefore, we measured the relative level of age pigments in adult nematodes at Day 10 after adulthood (about 13th day after hatching) to reflect the degree of physiological aging. As shown in Figure 5(a), the relative fluorescent intensity of age pigments in Day-10 nematodes was reduced by 23.2% and 42.7% after treatment with 2.0 and 4.0 mg/mL AsiPeps, respectively, as compared with the nematodes without peptide treatment, demonstrating that AsiPeps were capable of decreasing the accumulation of age pigments in aging nematodes. Interestingly, the wheat gluten hydrolyzate, a peptide-rich product, is recently shown to extend lifespan and decrease intestinal autofluorescence in* C. elegans* [40].

As lifespan is regarded as an unequivocal anti-aging index [22], we further investigated the effect of AsiPeps on the lifespan of* C. elegans* and showed that the lifespan of the nematodes was indeed extended after treatment with 2.0 and 4.0 mg/mL of the peptides (Figure 5(b)). Since the nematodes may cut down their consumption of food if a test sample is distasteful, dietary restriction, which is well known to retard development and delay aging in* C. elegans* [41], may contribute to the lifespan-extending effect of AsiPeps. To investigate this probability, extinction, which represents the optical density and internal structure, and TOF, which indicates the axial length of the nematodes, are measured to estimate nematode size and development [23, 42]. The pharyngeal pumping rate, which can be used to directly reflect food intake, was also determined. As shown in Figure 5(c), after treatment with a series of concentrations of AsiPeps (0.25–6.0 mg/mL), no obvious changes of extinction, TOF, and pharyngeal pumping rate were observed. Taken together, our data demonstrate that the* A. sinensis* peptides (AsiPeps) were able to extend the lifespan of* C. elegans* under physiological conditions, independent of dietary restriction.

## 4. Conclusion

In this paper, we isolated the peptides (AsiPeps) from* A. sinensis* protein hydrolyzate and demonstrate that AsiPeps were capable of not only improving oxidative survival of* C. elegans* models exposed to 70 mM paraquat but also decreasing endogenous ROS level, increasing antioxidant enzyme activities, and reducing lipid peroxidation product content of nematodes treated with 2 mM paraquat or undergoing senescence. AsiPeps were also shown to reduce age pigments content and extend lifespan of* C. elegans* but did not affect the food-intake behavior of the nematodes. Together, our data demonstrate that the* A. sinensis* peptides (AsiPeps) were able to delay aging in* C. elegans* through antioxidant activities but independent of dietary restriction.

## Figures and Tables

**Table 1:** Amino acid sequences of *A. sinensis* peptides identified by LC-MS/MS.

ID	Sequence	Number of residues	Molecular weight (Da)
1	DLTDFL	6	722.79
2	PSIVGRP	7	724.86
3	LTADILPR	8	898.07
4	QTVAVGVIK	9	914.11
5	AGLQFPVGR	9	944.10
6	AMPVEVVQF	9	1019.23
7	ESTAKQVIR	9	1031.18
8	VETGVIKPGM	10	1030.25
9	VVVNIPPTLK	10	1079.35
10	YGVSGYPTLK	10	1084.24
11	TTAGILLPEK	10	1042.24
12	AGFAGDDAPR	10	976.01
13	IGGIGTVPVGR	11	1025.22
14	GTIAGGGVIPH	11	978.12
15	GGVLPNINPVL	11	1092.30
16	AVFPSIVGRPR	11	1198.43
17	VLVSGSIHYPR	11	1227.43
18	TKMDEGVVTKK	11	1235.47
19	AAPFPGQKSLQR	12	1299.50
20	IKGTIAGGGVIPH	13	1219.45
21	LASSGIDHEGRLPR	14	1507.67
22	MIAFNKEQDTDLQSK	15	1767.98
23	IIGATNPAESAPGTIR	16	1567.76
24	EGGDGGYGGGGGGSRW	16	1425.39
25	EGGGGGYGGGGGGYGGR	17	1371.35
26	REGGGGGYGGGGGGYGGR	18	1527.53
27	GGGGYGGGGGGYGGGGGGY	19	1420.37
28	KKVGYNPDKIPFVPISGFEGDNMIER	26	2951.40

**Table 2:** The effect of *A. sinensis* peptides on the antioxidant enzyme activities and malondialdehyde content in *C. elegans* under oxidative stress. The nematodes were incubated with or without the peptides for 24 h prior to treatment with 2 mM paraquat.

Treatment	Antioxidant enzyme activity^a^		MDA content^b^
	SOD	CAT	
Control	31.44 ± 0.91	1.03 ± 0.01	42.54 ± 0.78
AsiPeps			
2.0 mg/mL	40.87 ± 0.75^c^	1.21 ± 0.02^c^	30.45 ± 1.49^c^
4.0 mg/mL	64.90 ± 1.24^c^	1.33 ± 0.01^c^	34.87 ± 1.34^c^

^a^SOD, U/mg protein; CAT, U/*μ*g protein; ^b^MDA, nmol/mg protein; ^c^ *p* < 0.05.

**Table 3:** The effect of *A. sinensis* peptides on the antioxidant enzyme activities and malondialdehyde content in *C. elegans* undergoing senescence. The data were determined at Day 10 after adulthood.

Treatment	Antioxidant enzyme activity^a^		MDA content^b^
	SOD	CAT	
Control	30.78 ± 0.53	2.51 ± 0.01	20.33 ± 1.69
AsiPeps			
2.0 mg/mL	32.54 ± 0.26^c^	2.57 ± 0.01	17.50 ± 1.05
4.0 mg/mL	47.13 ± 0.72^c^	3.00 ± 0.01^c^	15.82 ± 1.14^c^

^a^SOD, U/mg protein; CAT, U/*μ*g protein; ^b^MDA, nmol/mg protein; ^c^ *p* < 0.05.

## References

[1] Finkel T., Holbrook N. J. (2000). Oxidants, oxidative stress and the biology of ageing. *Nature*.

[2] Houstis N., Rosen E. D., Lander E. S. (2006). Reactive oxygen species have a causal role in multiple forms of insulin resistance. *Nature*.

[3] Dalle-Donne I., Rossi R., Milzani A., Di Simplicio P., Colombo R. (2001). The actin cytoskeleton response to oxidants: from small heat shock protein phosphorylation to changes in the redox state of actin itself. *Free Radical Biology and Medicine*.

[4] Yu L., Wan F., Dutta S. (2006). Autophagic programmed cell death by selective catalase degradation. *Proceedings of the National Academy of Sciences of the United States of America*.

[5] Chen W., Rezaizadehnajafi L., Wink M. (2013). Influence of resveratrol on oxidative stress resistance and life span in *Caenorhabditis elegans*. *Journal of Pharmacy and Pharmacology*.

[6] Abbas S., Wink M. (2009). Epigallocatechin gallate from green tea (*Camellia sinensis*) increases lifespan and stress resistance in *Caenorhabditis elegans*. *Planta Medica*.

[7] Xiao L., Li H., Zhang J. (2014). Salidroside protects *Caenorhabditis elegans* neurons from polyglutamine-mediated toxicity by reducing oxidative stress. *Molecules*.

[8] Kampkötter A., Pielarski T., Rohrig R. (2007). The *Ginkgo biloba* extract EGb761 reduces stress sensitivity, ROS accumulation and expression of catalase and glutathione S-transferase 4 in *Caenorhabditis elegans*. *Pharmacological Research*.

[9] Jiang H., Tong T., Sun J., Xu Y., Zhao Z., Liao D. (2014). Purification and characterization of antioxidative peptides from round scad (*Decapterus maruadsi*) muscle protein hydrolysate. *Food Chemistry*.

[10] Rajapakse N., Mendis E., Byun H.-G., Kim S.-K. (2005). Purification and in vitro antioxidative effects of giant squid muscle peptides on free radical-mediated oxidative systems. *Journal of Nutritional Biochemistry*.

[11] Chen N., Yang H., Sun Y., Niu J., Liu S. (2012). Purification and identification of antioxidant peptides from walnut (*Juglans regia* L.) protein hydrolysates. *Peptides*.

[12] Memarpoor-Yazdi M., Mahaki H., Zare-Zardini H. (2013). Antioxidant activity of protein hydrolysates and purified peptides from *Zizyphus jujuba* fruits. *Journal of Functional Foods*.

[13] Girgih A. T., He R., Malomo S., Offengenden M., Wu J., Aluko R. E. (2014). Structural and functional characterization of hemp seed (*Cannabis sativa* L.) protein-derived antioxidant and antihypertensive peptides. *Journal of Functional Foods*.

[14] Deng S., Chen S.-N., Yao P. (2006). Serotonergic activity-guided phytochemical investigation of the roots of *Angelica sinensis*. *Journal of Natural Products*.

[15] Lei T., Li H. F., Fang Z. (2014). Polysaccharides from *Angelica sinensis* alleviate neuronal cell injury caused by oxidative stress. *Neural Regeneration Research*.

[16] Guijarro-Díez M., García M. C., Marina M. L., Crego A. L. (2013). LC-ESI-TOF MS method for the evaluation of the immunostimulating activity of soybeans via the determination of the functional peptide soymetide. *Journal of Agricultural and Food Chemistry*.

[17] Guan S., Li P., Luo J. (2010). A deuterohemin peptide extends lifespan and increases stress resistance in *Caenorhabditis elegans*. *Free Radical Research*.

[18] Ranjan M., Gruber J., Ng L. F., Halliwell B. (2013). Repression of the mitochondrial peroxiredoxin antioxidant system does not shorten life span but causes reduced fitness in *Caenorhabditis elegans*. *Free Radical Biology and Medicine*.

[19] Burns A. R., Kwok T. C. Y., Howard A. (2006). High-throughput screening of small molecules for bioactivity and target identification in *Caenorhabditis elegans*. *Nature Protocols*.

[20] Gerstbrein B., Stamatas G., Kollias N., Driscoll M. (2005). *In vivo* spectrofluorimetry reveals endogenous biomarkers that report healthspan and dietary restriction in *Caenorhabditis elegans*. *Aging Cell*.

[21] Zhang H., Pan N., Xiong S. (2012). Inhibition of polyglutamine-mediated proteotoxicity by *Astragalus membranaceus* polysaccharide through the DAF-16/FOXO transcription factor in *Caenorhabditis elegans*. *The Biochemical Journal*.

[22] Wang Q., Yang F., Guo W. (2014). *Caenorhabditis elegans* in Chinese medicinal studies: making the case for aging and neurodegeneration. *Rejuvenation Research*.

[23] Smith M. V., Boyd W. A., Kissling G. E. (2009). A discrete time model for the analysis of medium-throughput *C. elegans* growth data. *PLoS ONE*.

[24] Panowski S. H., Wolff S., Aguilaniu H., Durieux J., Dillin A. (2007). PHA-4/Foxa mediates diet-restriction-induced longevity of *C. elegans*. *Nature*.

[25] Shalini S., Dorstyn L., Wilson C., Puccini J., Ho L., Kumar S. (2012). Impaired antioxidant defence and accumulation of oxidative stress in caspase-2-deficient mice. *Cell Death and Differentiation*.

[26] Rodriguez M., Basten Snoek L., De Bono M., Kammenga J. E. (2013). Worms under stress: *C. elegans* stress response and its relevance to complex human disease and aging. *Trends in Genetics*.

[27] Ayyadevara S., Bharill P., Dandapat A. (2013). Aspirin inhibits oxidant stress, reduces age-associated functional declines, and extends lifespan of *Caenorhabditis elegans*. *Antioxidants and Redox Signaling*.

[28] Wu Z., Ming J., Gao R. (2011). Characterization and antioxidant activity of the complex of tea polyphenols and oat *β*-glucan. *Journal of Agricultural and Food Chemistry*.

[29] Duthie G., Morrice P. (2012). Antioxidant capacity of flavonoids in hepatic microsomes is not reflected by antioxidant effects *in vivo*. *Oxidative Medicine and Cellular Longevity*.

[30] Pun P. B. L., Gruber J., Tang S. Y. (2010). Ageing in nematodes: do antioxidants extend lifespan in *Caenorhabditis elegans*?. *Biogerontology*.

[31] Sampayo J. N., Olsen A., Lithgow G. J. (2003). Oxidative stress in *Caenorhabditis elegans*: protective effects of superoxide dismutase/catalase mimetics. *Aging Cell*.

[32] Qian Z.-J., Jung W.-K., Byun H.-G., Kim S.-K. (2008). Protective effect of an antioxidative peptide purified from gastrointestinal digests of oyster, *Crassostrea gigas* against free radical induced DNA damage. *Bioresource Technology*.

[33] Zhang M., Mu T.-H., Sun M.-J. (2014). Purification and identification of antioxidant peptides from sweet potato protein hydrolysates by Alcalase. *Journal of Functional Foods*.

[34] Sampath Kumar N. S., Nazeer R. A., Jaiganesh R. (2012). Purification and identification of antioxidant peptides from the skin protein hydrolysate of two marine fishes, horse mackerel (*Magalaspis cordyla*) and croaker (*Otolithes ruber*). *Amino Acids*.

[35] Martorell P., Bataller E., Llopis S. (2013). A cocoa peptide protects *Caenorhabditis elegans* from oxidative stress and *β*-amyloid peptide toxicity. *PLoS ONE*.

[36] Labuschagne C. F., Stigter E. C. A., Hendriks M. M. W. B. (2013). Quantification of *in vivo* oxidative damage in *Caenorhabditis elegans* during aging by endogenous F3-isoprostane measurement. *Aging Cell*.

[37] Kim Y. S., Seo H. W., Lee M.-H., Kim D. K., Jeon H., Cha D. S. (2014). Protocatechuic acid extends lifespan and increases stress resistance in *Caenorhabditis elegans*. *Archives of Pharmacal Research*.

[38] Nielsen F., Mikkelsen B. B., Nielsen J. B., Andersen H. R., Grandjean P. (1997). Plasma malondialdehyde as biomarker for oxidative stress: reference interval and effects of life-style factors. *Clinical Chemistry*.

[39] Terman A., Brunk U. T. (2004). Lipofuscin. *International Journal of Biochemistry and Cell Biology*.

[40] Zhang W., Lv T., Li M. (2013). Beneficial effects of wheat gluten hydrolysate to extend lifespan and induce stress resistance in nematode *Caenorhabditis elegans*. *PLoS ONE*.

[41] Sutphin G. L., Kaeberlein M. (2008). Dietary restriction by bacterial deprivation increases life span in wild-derived nematodes. *Experimental Gerontology*.

[42] Moore B. T., Jordan J. M., Baugh L. R. (2013). WormSizer: high-throughput analysis of nematode size and shape. *PLoS ONE*.

